# The Treatment of Tonsils and Adenoids

**Published:** 1922-06

**Authors:** Thos. Clapham

**Affiliations:** Secretary, The Belgrave Hospital for Children. 1, Clapham Road, S.W.9.


*To the Editor of* The Hospital and Health Review.

### The Treatment of Tonsils and Adenoids.

*To the Editor of* The Hospital and Health Review.

Sir,—In your issue of May, 1922, there appears on page 223 a list of suggestions made by the Council of the Laryngological Section of the Royal Society of Medicine regarding the treatment of children suffering from tonsils and adenoids. A large number of children receive treatment for throat affections at this hospital, and in the past they have been treated in the out-patients' department as at other hospitals. The Committee of Management have given this matter special consideration during the last two years, and last autumn took the first step towards improving the conditions by providing an ambulance to convey the children to their homes instead of allowing them to travel in public vehicles shortly after the operation. The Committee have now decided to go much farther. On May 1 a special ward of ten beds was opened, and will be reserved entirely for the admission of throat cases. The children will be admitted the day before operation, and will stay in hospital three days and three nights, or longer if necessary.

I am,

Your obedient servant,

Thos. Clapham,

Secretary, The Belgrave Hospital for Children.

1, Clapham Road, S.W.9.

